# Correlation between private education costs and parental depression in South Korea

**DOI:** 10.1186/s12889-020-09058-w

**Published:** 2020-06-20

**Authors:** Byeong Cheol Oh, Ji-Yoon Yeon, Hyo-Sang Lee, Doo Woong Lee, Eun-Cheol Park

**Affiliations:** 1 College of Medicine, Medical Courses, Yonsei University, Seoul, South Korea; 2 Department of Public Health, Graduate School, Yonsei University, 50-1 Yonsei-ro, Seodaemun-gu, Seoul, 03722 South Korea; 3 Institute of Health Services Research, Yonsei University, Seoul, South Korea; 4 Department of Preventive Medicine & Institute of Health Services Research, Yonsei University College of Medicine, 50-1 Yonsei-ro, Seodaemun-gu, Seoul, 03722 South Korea

**Keywords:** Private education cost, Parental depression, Age, Equivalized household disposable income, Korea welfare panel study, Education-obsessed Korean society

## Abstract

**Background:**

In Korea, higher education has rapidly grown influenced by sociocultural tradition. Parents invest a significant portion of their household income in their children’s education. Private education has been considered to greatly affect students’ psychology and behavior. However, past research has largely neglected to study parents who pay these costs. Since household income and education level are important determinants of socioeconomic status (SES), education expenditures are likely to cause depressive symptoms. Therefore, the study aimed to investigate the correlation between private education costs and parental depression in South Korea.

**Methods:**

Data were collected from the Korean Welfare Panel Study (KoWePS, 2015, 2018). The sample analyzed consisted of 397 and 337 fathers and 403 and 370 mothers in 2015 and 2018, respectively. The independent variable in this study was the proportion of private education cost. This proportion was calculated by dividing each household’s private education costs by its equivalized household disposable income (EHDI) and multiplying this number by 100. The main dependent variable was parental responses to the Center for Epidemiologic Studies Depression Scale-11 (CESD-11). Using a generalized linear model, we investigated the effects of the proportion of private education cost on parental depression.

**Results:**

The results showed that fathers with higher proportions of private education cost exhibited higher CESD-11 scores compared to fathers with lower proportions cost (moderate: β = 0.419, S. E = 0.164, *p* = 0.0105; high: β = 0.476, S. E = 0.178, *p* = 0.0076), indicating that a higher ratio of private education cost may negatively affect depression in fathers. However, there was no discernable correlation between mothers’ CESD-11 scores and the proportion of private education cost (moderate: β = − 0.078, S. E = 0.250, *p* = 0.7555; high: β = 0.003, S. E = 0.215, *p* = 0.9882).

**Conclusions:**

These results may be explained by the tendency for fathers to experience greater economic burdens than mothers in patriarchal Korean society.

## Background

Depression is a widespread mental disorder all over the world. Korea is no exception. In Korea, about 6.7% of the total population suffers from depression [1]. In addition, a significant number of suicide deaths are closely related to depressive disorder [2]. South Korea has a high suicide rate, as it ranks first among the Organization for Economic Cooperation and Development (OECD) countries [3]. Therefore, it is necessary to find and address the factors that lead to depression.

Socioeconomic status (SES) is associated with an increased likelihood of depressive symptoms. Among the components of SES, household income is related to depressive symptoms, in both adults and adolescents [4, 5]. Academic success is closely related to social status, as education levels play an important role in defining the social class in Korean society [6]. Education levels reflect an important aspect of SES and are closely related to prestigious jobs [7]. Since household income and education level are important determinants of SES, education expenditures are likely to cause depressive symptoms.

Especially in Korea, higher education has grown rapidly with socio-cultural tradition (Confucian tradition), western educational ideas, and rapid economic growth [8]. Marginson proposes four elements that have developed educational needs in East Asia in relation to Confucian traditions: (1) strong nation-state policy (2) tertiary education funded by households (3) ‘one-chance’ national exams (4) public investment in research universities. These characteristics show that Confucian traditions have systematically influenced the development of higher education [9]. Koreans’ enthusiasm for education was an important factor not only to develop the national economy but also to expand higher education in the country [10]. In a strong desire to educate their children, parents invest a significant portion of their household income in their children’s education These phenomena have led to the development of education, but there have also been problems such as excessive education and shadow education [11, 12].

In South Korea, there is a strong interest in private education. Korean parents make substantial investments in private education to increase their children’s academic achievement. Parental obsession and dedication for their children’s educational and social success have been widely discussed in academic articles and media reports both in and outside Korea [13]. According to a Private Education Expenditures Survey 2018 conducted by Statistics Korea, the average monthly private education expenses per student in Korea is 291,000 Won (about 249 dollars) and it is generally increasing every year. If the figure is adjusted to account for 73% of students, excluding those without private education costs, spending increases to 399,000 Won (about 342 dollars) per student. The total cost of private education in 2018 increased by 4.4% compared to last year. Private education costs in Korea are high enough to account for 7.7% of households’ total consumption expenditure [14]. Since households’ total consumption expenditure also includes households without children, the proportion of private education costs for households with children should be higher. While many students are receiving private education to improve their academic performance, private education costs differ by household income. According to a Private Education Expenditures Survey 2018, the higher the average monthly household income, the higher the private education costs and participation rate [14]. Since private education affects a student’s academic performance, parents could be concerned about private education and household income. Thus, it is crucial to understand how private education affects households. Affected targets are largely divided into children and parents.

Private education in South Korea has adverse effects from excessive parental urgency, private education costs, and private tutoring methods [15]. There have been some studies on how private education has affected students. Private tutoring increase student’s academic stress and the increase in private education is likely to lead to depression in the children of the poor due to the burden of education costs, poor academic performance, academic achievement, and stress [16, 17]. The previous study has also shown that participation time in private education has an indirect impact on depression through academic stress and academic achievement [17]. The link between student depression and parental depression has also been analyzed in earlier research [18].

However, there are not enough amount of studies on how private education affects parents. Most extant studies exploring the relationship between private education and parents focus on parent’s participation in education [19–21]. In addition to the economic burden, private education is a burden for parents in the process of checking better private tutoring options and interacting with private tutors [20, 22]. As demonstrated before, household income and education level are important determinants of SES, so education expenditures are likely to cause depressive symptoms. Therefore, this study aimed to investigate the correlation between private education costs and parental depression in South Korea.

## Methods

### Design

We collected data from the 2015 and 2018 outcomes of the Korean Welfare Panel Study (KoWePS), which is a nationwide annual study that provides reliable data on Korean households. Approximately 50% of the KoWePS sample consisted of individuals in low-income brackets with less than 60% median income; thus, the survey was for low-income policy and poverty research. The survey investigated household information (general information, health, medical care, economic status, social insurance, retirement pension, housing, living conditions, living expenses, and income) and household member information (social insurance, labor, living conditions, private education, and mental health). Since a supplementary children’s survey was administered to the same respondents in a three-year cycle, we used data from 2015 and 2018 for families with children in elementary school 4th–6th grades in 2015 and middle school 1st–3rd grade in 2018 according to Korean education course. We built our dataset by linking parent survey responses with household income and children’s survey responses.

### Participants

**Fig. 1:**
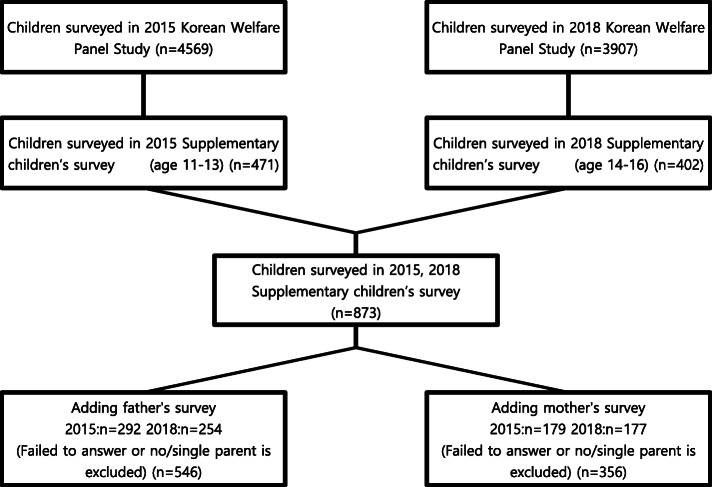
Flow diagram displaying the inclusion and exclusion of subjects

The KoWePS 2015 and 2018 surveyed 13,647 and 12,469 participants, respectively. The number of children surveyed in 2015 and 2018 with the Supplementary Children’s Survey was 471 and 402, respectively. We added parental response data to the children’s response data table and separated the fathers’ and mothers’ variables to test the hypothesis. Since men are usually financially responsible in Korea society, father and mother were studied separately [23]. Each child’s information was combined separately with their fathers’ and mothers’ information in two distinct analyses. The following participants’ data were excluded. First, children with only one parent were excluded, in order to eliminate the impact of differences in financial burdens. Second, participants who failed to complete the questionnaires were also excluded. The analyzed sample consisted of 292 and 254 fathers in 2015 and 2018 and 179 and 177 mothers in 2015 and 2018, respectively, as seen in Fig. 1.

### Variables

In this study, the independent variable was the proportion of private education cost, which was calculated by dividing household private education costs by the EHDI (Equivalized household disposable income) and multiplying this number by 100.

$$ The\ proportion\ of\ private\ education\  co\mathrm{s}t=\frac{Private\ education\ cost}{Equivalized\ household\ disposable\ income}\times 100 $$

Disposable income is one of the main indicators used to measure the overall economic status of households, and it is the widely used indicator of welfare level [24]. To calculate disposable income, market income and public transfer income were summed, and public transfer expenditure was subtracted [25, 26].

$$ Market\ income+ Public\ transfer\ income- Public\ transfer\ expenditure= Household\ disposable\ income. $$

EHDI was calculated using the following formula because changes in the number of household members can affect results. This formula provides an adequate comparison of disposable income between various members and households [25–27].

$$ \mathrm{E} quivalised\ Household\ disposable\ income=\frac{\  Household\ disposable\ income}{\sqrt{Number\ of\ household\ member(s)}} $$

After calculating the proportion of private education cost, we divided the sample into three groups (low, mid, or high) according to the tertiles.

The main dependent variable was parental responses to the Center for Epidemiologic Studies Depression Scale-11 (CESD-11). The Center for Epidemiological Studies Depression Scale (CESD) developed by Radloff was originally a 20-item self-report index of depressive symptoms designed for the use in large-scale surveys. CESD is a reliable and well-validated instrument for screening depression signs and symptoms in the elderly of all kinds of linguistic, racial, aging, and health status cohort of the population [28, 29]. CESD has four relatively invariant factors that are readily interpretable as Depressed Affect (being blues, depressed, lonely, failure, fearful, cry, or sad); Positive Affect (as good as others, felt hopeful, happy, or enjoyed life); Somatic Problems (having been bothered, appetite, trouble, effort, sleep, talked less, or could not get going); and Interpersonal Problems (feeling unfriendly, or dislike) [30].

Parental depression was measured using the CESD-11, a shortened version of the 20-item CESD. The factor structure of CESD-11 has the same four factors as those in the original CESD [31]. According to a survey conducted by KoWePS, Depressed Affect includes item 3, 6, and 9; Positive Affect includes item 2 and 7; Somatic Problems includes item 1, 4, 5, and 11; and Interpersonal Problems includes item 8 and 10. Response categories were ‘① Rarely or none of the time (Less than 1 day in last week) ‘, ‘② Some of the time (1–2 days in last week) ‘, ‘③ Much of the time (3–4 days in last week)’, and ‘④ Most or all the time (5-7 days in last week). The score was constructed following the guidelines of KoWePS. The number of the response became the score of each item. For Positive Affect (item 2, 7), the score was converted into ‘5-number’ (scores: 1 → 4, 2 → 3, 3 → 2, 4 → 1). The total score was calculated by adding the scores for all questions and multiplying the value by 20/11. Depression was diagnosed in individuals who reported CESD-11 scores above 16 [32]. The values of Cronbach’s α are 0.81 for CESD-11 compare to 0.86 for CESD [33]. Supplementary Table 1 provides basic statistics for participants’ CESD-11. Statistics show that the father’s depression has a positive correlation with the proportion of private education cost, and the father’s depression in 2018 is slightly higher than in 2015.

Other factors affecting parental depression were also assessed. Parents’ socio-demographic factors included age, private educational level (no high school private education or high school academic background), and residence (suburban, urban). Economic variables included household income (low, mid, or high, according to the tertiles) and economic activity (employed and unemployed). Health-related factors included chronic disease (none, diagnosed) and alcohol usage disorder identification test (AUDIT) score (WHO). Psychological factors included the Rosenberg Self-Esteem Scale (RSS) score, cost satisfaction (very low, low, normal, high, and very high), family satisfaction (very low, low, normal, high, and very high), and child satisfaction (very low, low, normal, high, and very high). Factors related to children included children’s grade point average (GPA) (very low or low, normal, and high or very high), children’s study stress (adding the scores of 4 questions with 4 point scale and grouping the points according to the tertiles into low, mid, or high), and children’s Korean Child Behavior Checklist (K-CBCL) scores (low, mid, or high, according to the tertiles) (Rosenberg, 1965; Oh, Lee, Hong, & Ha, 1997). For Table 3, parent’s age grouping was decided according to the tertiles (low, mid, or high) of each year (the dividing point of 2018 is calculated by adding 3 to the dividing point of 2015).

### Statistical analysis

To analyze the association between parental depression and the proportion of private education cost, we performed a multiple regression analysis after controlling for covariates. These covariates included the household income, parental age, private education level, working status, residence, RSS scores, AUDIT scores, chronic disease, cost satisfaction, family satisfaction, child satisfaction, children’s GPA, children’s study stress, and children’s K-CBCL scores. Finally, we performed subgroup analyses for the association between the proportion of private education cost and other listed factors, according to parental depression, after adjusting for covariates. Data were analyzed using a log-linear Poisson regression model (SAS Genmod procedure, version 8.1) with generalized linear models. *P*-values less than 0.05 were considered statistically significant.

## Results

Table 1 includes the general characteristics of the study sample after excluding the missing values. Of the sample comprising 292 fathers, the mean CESD score for a low proportion of private education cost (32.2%) was 2.71. The mean CESD score for a moderate proportion of private education cost (34.6%) was 3.46. The mean CESD score for a high proportion of private education cost (33.2%) was 3.66. Of the 179-person mother sample, the mean CESD score for a low proportion of private education cost (31.8%) was 3.03. The mean CESD score for a moderate proportion of private education cost (31.8%) was 3.03. The mean CESD score for a high proportion of private education cost (36.3%) was 3.02.

**Table 1:** General characteristics of study subjects at 2015 baseline year

Variables	Fathers’ CESD					Mothers’ CESD				
	Total	%	Mean	SD	*p-value*	Total	%	Mean	SD	*p-value*
Total	292	100.0	3.28	6.26		179	100.0	3.03	5.85	
**Proportion of private education cost**^**a**^										
Tertile 1 (0~16.3%/0~17.5%)	94	32.2	2.71	5.06	.2763	57	31.8	3.03	5.32	.9999
Tertile 2 (16.3~29.4%/17.5~29.0%)	101	34.6	3.46	6.18		57	31.8	3.03	6.74	
Tertile 3 (29.4%~/29.0%~)	97	33.2	3.66	7.33		65	36.3	3.02	5.52	
**EHDI**^**b**^										
Tertile 1 (~198.0/~202.4)	135	46.2	4.26	7.84	.0018	84	46.9	4.11	7.04	.0103
Tertile 2 (198.0~278.5/202.4~283.3)	75	25.7	2.50	5.00		51	28.5	1.89	3.88	
Tertile 3 (278.5~/283.3~)	82	28.1	2.39	3.66		44	24.6	2.27	4.88	
**Age**			45.78	4.20				42.83	3.75	
**Education**										
No high school education	10	3.4	6.73	7.33	.0104	4	2.2	3.18	6.36	.9442
High school academic background	282	96.6	3.16	6.20		175	97.8	3.02	5.85	
**Work**										
No	6	2.1	4.55	3.94	.4662	65	36.3	3.55	6.04	.2371
Yes	286	97.9	3.25	6.30		114	63.7	2.73	5.74	
**Residence**										
Suburban	29	9.9	3.01	4.82	.7191	14	7.8	2.99	3.81	.9723
Urban	263	90.1	3.31	6.41		165	92.2	3.03	5.99	
**Chronic disease**										
None	215	73.6	2.90	5.97	.0119	151	84.4	2.30	4.07	<.0001
Diagnosed	77	26.4	4.34	6.94		28	15.6	6.95	10.68	
**Child's GPA**										
Low	17	5.82	6.84	13.94	.0016	10	5.59	5.64	8.46	.1348
Middle	83	28.42	3.42	6.05		48	26.82	2.50	4.40	
High	192	65.75	2.91	5.14		121	67.60	3.02	6.09	
**Child's study stress**										
Tertile 1 (0~6/0~6)	115	39.4	3.23	6.22	.1080	72	40.2	1.79	4.35	.0095
Tertile 2 (6~8/6~9)	80	27.4	2.57	4.43		71	39.7	3.64	5.71	
Tertile 3 (8~12/9~12)	97	33.2	3.94	7.48		36	20.1	4.29	8.04	
**Child's K-CBCL**										
Tertile 1 (0~5/0~5)	103	35.3	2.82	6.05	.0337	58	32.4	2.76	5.00	.2330
Tertile 2 (5~12/5~13)	94	32.2	2.82	4.64		64	35.8	3.78	7.76	
Tertile 3 (12~/13~)	95	32.5	4.23	7.68		57	31.8	2.46	3.79	
**RSS**			22.53	2.46				22.48	2.15	
**AUDIT**			9.15	5.32				4.27	3.25	
**Fee satisfaction**			3.72	0.76				3.79	0.66	
**Family satisfaction**			4.86	1.15				4.83	1.14	
**Child satisfaction**			2.80	0.42				2.79	0.46	

Abbreviations: *CESD* (The Center for Epidemiologic Studies Depression Scale), *EHDI* (Equivalized Household Disposable Income), *AUDIT* (Alcohol Usage Disorder Identification Test), *RSS* (Rosenberg Self-Esteem Scale), *GPA* (Grade Point Average), *K-CBCL* (Korean Child Behavior Checklist)

^1^Proportion of private education cost is defined as: (private education cost/equivalized household disposable income)*100

^2^The unit of EHDI is **₩**10,000 which is about $8.83 at 2015

In the parentheses, the first part is the father’s actual data range and the second part is the mother’s

Table 2 presents the results of the multiple regression model used to analyze the factors associated with parent’s depression as measured by the CESD. Fathers with higher proportions of private education cost reported higher CESD scores when compared to those with lower proportions of private education cost (moderate: β = 0.419, S. E = 0.164, 95%CI = 0.098 ~ 0.740, *p* = 0.0105; high: β = 0.476, S. E = 0.178, 95%CI = 0.126 ~ 0.825, *p* = 0.0076). For mothers, however, the results revealed that there was no correlation between CESD scores and the proportions of private education cost (moderate: β = − 0.078, S. E = 0.250, 95%CI = − 0.568 ~ 0.412, *p* = 0.7555; high: β = 0.003, S. E = 0.215, 95%CI = − 0.418 ~ 0.425, *p* = 0.9882).

**Table 2:** Association between proportion of private education cost to EHDI and depression among parents

Variables	Fathers’ CESD				Mothers’ CESD			
	β	SE	95% CI	*p-value*	β	SE	95% CI	*p-value*
**Proportion of private education cost**^**a**^								
Tertile 1 (0~16.3%/0~17.5%)	Ref.				Ref.			
Tertile 2 (16.3~29.4%/17.5~29.0%)	0.419	0.164	0.098~0.740	.0105	-0.078	0.250	-0.568~0.412	.7555
Tertile 3 (29.4%~/29.0%~)	0.476	0.178	0.126~0.825	.0076	0.003	0.215	-0.418~0.425	.9882
**EHDI**^**b**^								
Tertile 1 (~198.0/~202.4)	Ref.				Ref.			
Tertile 2 (198.0~278.5/202.4~283.3)	-0.040	0.200	-0.431~0.351	.8415	-0.385	0.212	-0.801~0.030	.0692
Tertile 3 (278.5~/283.3~)	-0.330	0.180	-0.682~0.022	.0662	-0.383	0.280	-0.932~0.166	.1713
**Age**	0.058	0.018	0.022~0.093	.0015	0.020	0.038	-0.054~0.094	.5973
**Education**								
No high school education	-0.207	0.313	-0.821~0.407	.5084	-0.217	0.401	-1.004~0.569	.5880
High school academic background	Ref.				Ref.			
**Work**								
No	-0.112	0.395	-0.886~0.662	.7763	0.077	0.184	-0.285~0.438	.6775
Yes	Ref.				Ref.			
**Residence**								
Suburban	0.294	0.209	-0.115~0.704	.1590	-0.149	0.328	-0.792~0.494	.6496
Urban	Ref.				Ref.			
**Chronic disease**								
None	0.163	0.136	-0.103~0.429	.2306	-0.522	0.168	-0.851~-0.192	.0019
Diagnosed	Ref.				Ref.			
**RSS**	-0.021	0.042	-0.103~0.061	.6191	0.062	0.046	-0.027~0.151	.1713
**AUDIT**	0.042	0.013	0.016~0.068	.0017	0.020	0.030	-0.038~0.078	.4946
**Fee satisfaction**	-0.592	0.089	-0.767~-0.417	<.0001	-0.452	0.098	-0.643~-0.261	<.0001
**Family satisfaction**	-0.078	0.072	-0.220~0.064	.2805	-0.097	0.077	-0.248~0.055	.2109
**Child satisfaction**	-0.478	0.145	-0.762~-0.194	.0010	-0.392	0.165	-0.714~-0.069	.0172
**Child's GPA**								
Low	0.278	0.245	-0.203~0.758	.2569	-0.030	0.330	-0.676~0.617	.9280
Middle	0.218	0.143	-0.061~0.498	.1260	0.086	0.180	-0.267~0.439	.6322
High	Ref.				Ref.			
**Child's study stress**								
Tertile 1 (0~6/0~6)	0.085	0.160	-0.229~0.399	.5959	-0.753	0.270	-1.283~-0.224	.0053
Tertile 2 (6~8/6~9)	0.025	0.177	-0.322~0.371	.8889	-0.203	0.205	-0.606~0.199	.3228
Tertile 3 (8~12/9~12)	Ref.				Ref.			
**Child's K-CBCL**								
Tertile 1 (0~5/0~5)	-0.114	0.181	-0.469~0.242	.5309	0.188	0.210	-0.223~0.598	.3704
Tertile 2 (5~12/5~13)	-0.059	0.193	-0.437~0.318	.7579	0.320	0.221	-0.114~0.753	.1486
Tertile 3 (12~/13~)	Ref.				Ref.			

Abbreviations: *CESD* (The Center for Epidemiologic Studies Depression Scale), *EHDI* (Equivalized Household Disposable Income), *AUDIT* (Alcohol Usage Disorder Identification Test), *RSS* (Rosenberg Self-Esteem Scale), *GPA* (Grade Point Average), *K-CBCL* (Korean Child Behavior Checklist)

^1^Proportion of private education cost is defined as: (private education cost/equivalized household disposable income)*100

^2^The unit of EHDI is **₩**10,000 which is about $8.83 at 2015

In the parentheses, the first part is the father’s actual data range and the second part is the mother’s

Table 3 presents the results of the subgroup multiple regression analyzing the association between parent’s depression and proportions of private education cost according to EHDI. Higher proportions of private education cost significantly increased depression among fathers who lived in households with low EHDI (moderate: β = 1.172, S. E = 0.365, 95%CI = 0.457 ~ 1.887, *p* = 0.0013; high: β = 1.437, S. E = 0.313, 95%CI = 0.823 ~ 2.051, *p* < 0.001). There is a blank space in the table because the model did not converge.

**Table 3:** Results of subgroup analysis by proportion of private education cost to CESD scores according to EHDI

Variables	Proportion of private education cost^a^								
	Tertile 1 (~16.3%/~17.5%)	Tertile 2 (16.3~29.4%/17.5~29.0%)				Tertile 3 (29.4%~/29.0%~)			
	β	β	SE	95% CI	*p*-value	β	SE	95% CI	*p*-value
**Father**									
**EHDI**^**b**^									
Tertile 1 (~198.0)	Ref.	1.172	0.365	0.457~1.887	.0013	1.437	0.313	0.823~2.051	<.0001
Tertile 2 (198.0~278.5)	Ref.	-0.070	0.235	-0.532~0.391	.7650	0.013	0.210	-0.398~0.424	.9492
Tertile 3 (278.5~)	Ref.	0.352	0.216	-0.071~0.776	.1032	-0.132	0.295	-0.711~0.446	.6535
**Mother**									
**EHDI**^**b**^									
Tertile 1 (~202.4)	Ref.	-0.259	0.355	-0.956~0.437	.4654	-0.150	0.340	-0.817~0.517	.6593
Tertile 2 (202.4~283.3)	Ref.	-0.236	.	.	.	-0.134	.	.	.
Tertile 3 (283.3~)	Ref.	-0.303	0.319	-0.928~0.322	.3416	-0.169	0.333	-0.821~0.483	.6120

Abbreviations: *CESD* (The Center for Epidemiologic Studies Depression Scale), *EHDI* (Equivalized Household Disposable Income), *AUDIT* (Alcohol Usage Disorder Identification Test), *RSS* (Rosenberg Self-Esteem Scale), *GPA* (Grade Point Average), *K-CBCL* (Korean Child Behavior Checklist)

^1^Proportion of private education cost is defined as: (private education cost/equivalized household disposable income)*100

^2^The unit of EHDI is **₩**10,000 which is about $8.83 at 2015

Value in the parentheses is the actual data range

## Discussion

This study revealed that as the proportion of private education cost changes, it affects the fathers’ depression levels but not that of the mothers. This suggests that fathers are mostly concerned about their children’s private education costs. Family roles in Korea often dictate fathers to offer financial resources thus they experience greater economic burdens than mothers. Although gender roles are changing, men have historically been expected to earn money while women are expected to perform childcare and housekeeping activities. This patriarchal system remains in Korea [34]. According to research participants, while only 2.1% of the total number of fathers were unemployed, as many as 36.3% of the total number of mothers were unemployed. This demonstrates that fathers bear a greater responsibility to earn money. In South Korea, under the Confucian tradition, father-children relationships are highly valued. Traditional fatherhood is based on the use of discipline and guidance in children’s education and not on warmth or care [35]. However, these traditional family ideals are challenged as society changes. New paternal expectations demand parents spend more time with their kids [36]. Nevertheless, it is likely that many working-class fathers are still after the Confucian ideals. Those fathers do not spend time with their children but only care about their child’s academic performance [37]. As our research data was collected from KoWePS, the participants were mostly working-class families. Participants would mostly follow Confucian ideals and care about a child’s academic performance. In Korea, the responsibility for children’s education is largely left to the mother, so the mother can be stressed from private education [38]. But the stress comes from educating children, not spending on private tuition. While the mother decides how to use private education, it is a father who feels burdened with the spending. However, as Confucianism is gradually disappearing in Korea, dual-income families are increasing [39]. The economic burden, which has traditionally belonged to men, will gradually become a burden to both parents.

Numerous studies have investigated the impact of family resources on educational performance [40]. While parents invest in their children’s education, these investments incur opportunity costs for other investments that parents or their families want [41]. Private education is a potential investment for children. Therefore, parents should evaluate this investment against other important expenditures [42]. Becker provides the intrafamily decision-making model, in which the household acts as one economic agent that optimizes a multiperiod utility function. Investment in the child comes at the opportunity cost of present-period consumption by the parent [43]. Numerous researchers have applied the Becker model to their studies to investigate the economics of parenting [40, 44]. Studies suggest that there are intergenerational conflict and moral risks in the household which leads to an intrahousehold dilemma. These financial worries may be deeply associated with depression and anxiety [45]. It is obvious that lower-income households are more sensitive to the proportion of private education costs than those with higher levels of income. (see Table 3).

A previous study of private education revealed that private education negatively affects children’s academic stress [46]. In Korea’s contemporary education-obsessed society, private education costs significantly affect not only children but families as a whole. High private education costs not only affect the mental health of the family but also low birth rates and educational inequality. When parents decide how many children to raise, they consider the cost of raising children and their economic strength so that all their children have a good capacity [47]. Inequality of educational opportunity due to the socioeconomic status of parents makes a difference in the educational attainment of the children’s generation [48].

Private education refers to supplementary education in addition to public education. Therefore, it poses a risk of educational inequality. Korean students study hard to attend highly reputed tertiary schools [49]. In 2018, 69.7% of high school graduates entered higher education institutions, which is 65.5% of the population of the same age [50]. It could be a positive social phenomenon in that many people receive higher education, but this leads to excessive private education spending. Parents do not want their children to fall behind, so they rely heavily on private education. Parents aware that future income depends on the education level. According to the Korea Statistics 2018, education level and hourly wages are highly correlated [50]. Parents make their children study, hoping that they will be richer than they are, but the level of education already depends on their financial level. Inequality drives parents into an endless competition, but this competition only results in intensified inequality [51]. Educational inequality is one of the fundamental causes of private educational use.

This study focused on parental depression caused by private education, while many other studies focused on children’s mood. Also, using the socio-cultural contexts of Korea, the reasons why fathers are more likely to be affected by higher proportions of private education cost, and why private education is highly activated are explained. Our findings argue that the impact of private education on parents should be highly considered. Recently, there has been much discussion about parenting in economics [52]. Parenting is affected by sociocultural contexts, economics, and thus affects parent mental health. Therefore, the proportions of private education cost could serve as an important variable in familial economic decision-making. Also, research on private education costs may give a deeper understanding of parenting. However, this study has some limitations. By using the given data from KoWePS, only researched variables were available. If the number of panels was bigger, a better understanding of the findings could be possible. CESD assesses short-term depressive mood, so it may not reflect the long-term associations which lead to clinical depression. Since the children in the study group included only students aged 11–16, the generalization of the results may be limited. In addition, the survey was conducted by KoWePS, which is suitable for low-income policies and poverty studies, so the generalization of different social statuses may be limited. However, since it was investigated by the government, the data is reliable.

## Conclusions

The results of this study show an association between the proportion of private education cost and fathers’ depression levels in Korea. Korean traditional Confucian ideologies affect fathers to burden private education costs. As private education cost comes from the opportunity cost of consumption for household, raising private education costs, and lowering household incomes lead to parental depression. Since high private education costs affect families as a whole, the government should attempt to address private education problems. The government announced measures to reduce private education and normalize public education in 2014 [53]. However, these efforts were not enough, and the issue remains prevalent. In response, the government should attempt to address more fundamental problems. A social policy that guarantees employment opportunities through compulsory education will be the fundamental solution to these private education cost problems. A society that can provide quality jobs through vocational training after compulsory education will reduce private education for compulsory education. Education inequality will be alleviated if the government strengthens public education and provides educational subsidies to reduce the cost of private education [54]. The process may be challenging, but it can ease the burden on low-income families.

## Supplementary information

**Additional file 1: Table S1.** Comparison of CESD according to year and proportion of private education cost. Abbreviations: CESD (The Center for Epidemiologic Studies Depression Scale). ^1^Proportion of private education cost is defined as: (private education cost/equivalized household disposable income)*100. Value in the parentheses is the actual data range.

## Data Availability

The dataset analyzed during the current study are available in the Korea Welfare Panel Study, https://www.koweps.re.kr:442/.

## Abbreviations

KoWePS: Korea Welfare Panel Study
CESD: The Center for Epidemiologic Studies Depression Scale
EHDI: Equivalized Household Disposable Income
AUDIT: Alcohol Usage Disorder Identification Test
RSS: Rosenberg Self-Esteem Scale
K-CBCL: Korean Child Behavior Checklist
